# Salience of self-identification of transsexual people in different stages of medical transition

**DOI:** 10.1192/j.eurpsy.2021.1463

**Published:** 2021-08-13

**Authors:** S. Kumchenko, A. Tkhostov, E. Rasskazova

**Affiliations:** 1 Faculty Of Psychology, Lomonosov MSU, Moscow, Russian Federation; 2 Clinical Psychology, Moscow State University, Moscow, Russian Federation

**Keywords:** self-identification, transsexuals, Transgender, medical transition

## Abstract

**Introduction:**

Transsexuals are considered to be stable in their identity (White Hughto et al., 2016). Meanwhile, the stages of medical transition affect the mental state of transsexuals differently.

**Objectives:**

The aim was to reveal relationships between salience of self-identification in transsexual people being on different stages of medical transition.

**Methods:**

151 transsexual people: 55 pre-operated Female-to-Male (FtM I), 25 FtM on a hormonal therapy (FtM II), 25 FtM after some surgical operations (FtM III); 12 pre-operated Male-to-Female-Transsexual (MtF I), 16 MtF on a hormonal therapy (MtF II), 18 MtF after some surgical operations (MtF III). The participants filled the modificated Kuhn’s test “Who am I?” (Tkhostov et al., 2014). The modification includes a Likert scale for evaluating one’s self-identifications in terms of salience: “How often do You think or remember this answer?” (Stryker, 2007).

**Results:**

There were differences between identity salience and stages of medical transition (F = 7,177; P < 0,001; η2 = 0,108). Transsexuals before medical transition demonstrated higher levels of identity salience (average score is 7,62 in FtM I and 7,75 in MtF I). Transsexuals on a hormonal therapy demonstrated sharply decreased level of identity salience (6,97 in FtM II and 6,19 in MtF II). Transsexuals after surgical operations reported increased level of salience (7,81 in FtM III and 7,23 in MtF III). There were no statistically significant differences between the groups by gender assigned at birth.

**Conclusions:**

Data suggest that medical transition could change the salience of self-identification. Hormone therapy is associated with a sharp revision of the salience of self-identifications for transsexuals.

